# Legislation in New York State

**Published:** 1896-04

**Authors:** 


### VETERINARY LEGISLATION IN NEW YORK STATE.

IN 1886 the initial step in veterinary legislation was taken, the first law requiring registration being passed that year. At various periods this law has been amended, and the time for registration extended until the passage, last year, of the Regents' Bill, when we thought that the possibility of further time-extension was *nil.* We were surprised not long since to find that a bill had been introduced and passed in the Assembly to permit a certain person to register within a prescribed time; and, a little later on, we were simply amazed when Mr. Harrison introduced a bill, which, if it becomes a law, will give a certain James Wixon, of Steuben County, the legal right to practise veterinary medicine and surgery as a profession.

The bill exempting veterinarians of this State from jury-duty passed some time ago, and we congratulated ourselves that we need no more be bothered by having to serve as jurors, when to our surprise we were peremptorily summoned, being told that the bill, as passed, exempted from such service all the profession of this State with the exception of New York and Kings County. This was indeed discouraging. On making further inquiries we found that the Act, as passed, was an amendment to another law that exempted medical men in all the counties but New York and Kings, but, unfortunately, this omission was not discovered until too late. This, of course, necessitated the preparation and introduction of another bill, exempting us from jury-duty in the two counties mentioned.

With the jury, registration-extension, and the James Wixon special bill hung up in committee, we must say that at present the veterinary legislation of New York State is in a deplorable condition.

The Jury Bill should become a law, and we admonish every practitioner to use his personal influence in the furtherance of the same; but still, rather than permit the two last-mentioned bills to become laws, we could cheerfully endure the inconvenience, loss of time, etc., by having to serve as jurors.

What right has James Wixon, or any other man, to ask for legislation to qualify him legally, when we have the Regents' law, which gives him an opportunity either to register by affidavit or pass the examination? If too obstinate to register within the time limited he does not deserve to be allowed to practise; if too ignorant to pass the Regents' examination he is not a fit subject to enter the ranks of the veterinary profession, and, to say the least, would be an imposition on the public.

